# Lethal impacts of cigarette smoke in cultured tobacco cells

**DOI:** 10.1186/1617-9625-9-8

**Published:** 2011-07-16

**Authors:** Masaru Yukihiro, Takuya Hiramatsu, Tomonori Kawano

**Affiliations:** 1 Laboratory of Chemical Biology and Bioengineering, Faculty and Graduate School of Environmental Engineering, The University of Kitakyushu, Kitakyushu 808-0135, Japan

## Abstract

**Background:**

In order to understand and generalize the toxic mechanism of cigarette smoke in living cells, comparison of the data between animal systems and other biological system such as microbial and plant systems is highly beneficial.

**Objective:**

By employing the tobacco cells as model materials for cigarette smoke toxicity assay, the impacts of the combustion by-products such as nitrogen oxides could be highlighted as the toxic impacts of the plant-derived endogenous chemicals could be excluded in the plant cells.

**Methods:**

Cigarette smoke-induced cell death was assessed in tobacco cell suspension cultures in the presence and absence of pharmacological inhibitors.

**Results:**

Cigarette smoke was effective in induction of cell death. The smoke-induced cell death could be partially prevented by addition of nitric oxide (NO) scavenger, suggesting the role for NO as the cell death mediator. Addition of NO donor to tobacco cells also resulted in development of partial cell death further confirming the role of NO as cell death mediator. Members of reactive oxygen species and calcium ion were shown to be protecting the cells from the toxic action of smoke-derived NO.

## Introduction

In the United States, the toxic impacts of various chemicals to various organisms have been documented in the Ecotoxicology Database (ECOTOX) of the US EPA. The cigarette smoke is known to be toxic and thus harmful to human health [1], both at cellular [2] and genetic levels [3]. On the other hand, the impacts of cigarette smoke in various organisms including living plants have been poorly documented to date. In order to understand and generalize the toxic mechanism of cigarette smoke in living cells, comparison of the data between animal systems and other biological system such as microbial and plant systems is highly beneficial.

Since the cigarette smoke is derived from combustion of tobacco leaves, the chemical components in the smoke must be the mixture of (i) chemical contents originally present in the tobacco leaves and (ii) the chemicals formed through combustion process (combustion by-products) [4]. Both former (such as nicotine, phenolics, *etc*.) and latter chemicals (such as hydrogen peroxide) are known to be harmful to human health [3]. However, it is natural to assume that former group of chemicals such as nicotine should not be toxic to living tobacco cells since tobacco cells are naturally rich in those chemicals (as genetically equipped with genes required for biosynthesis of nicotine [5] and phenolics [6]). Therefore, by employing the tobacco cell lines as model materials for cigarette smoke toxicity assay, we can exclude the toxic impacts of the plant-derived endogenous chemicals but the impacts of the combustion by-products such as NO_x _could be highlighted. Use of plant materials as the models for human health is not surprisingly unusual. In fact, many discoveries with direct relevance to human health and disease have been elaborated using plant models, and several processes important to human biology are more easily studied in these versatile model plants [7].

In addition to native plant-derived compounds and smoke-derived combustion by-products, *de novo *synthesis of toxic chemicals such as reactive oxygen species (ROS), chiefly superoxide within the human cells exposed to cigarette smoke has been documented [8]. Thus, our interest covered the role of ROS in smoke-induced cell death in living tobacco cells. Here, cigarette smoke-induced cell death in tobacco cell culture was assessed and a possible key smoke component involved in lethal mechanism was determined. Lastly, through discussion, the mechanism found in plant system was compared with those found in animal system.

## Methods

### Cigarettes

As model cigarettes, packs of filter-equipped cigarettes "MILD SEVEN Light" (Japan Tobacco Inc., Tokyo, Japan) were purchased from a local vender.

### Plant materials

Two suspension-cultured cell lines of tobacco (*Nicotiana tabacum *L.) derived from the Bel-W3 variety which is highly sensitive to oxidants such as ozone and reactive oxygen species (ROS) and the Bel-B variety which is highly tolerant to ozone and related ROS, were used as model materials.

As previously described [9], the suspension cultures of Bel-W3 and Bel-B cells (5 day-old culture) were propagated in the Murashige-Skoog (MS) liquid medium (pH 5.8) containing 0.2 μg/ml of 2,4-D. The cell suspension cultures (30 ml each in a 100 ml conical flask) were kept on gyratory shakers (at 130 rpm) at 23 ± 1°C in darkness, with occasional sub-culturing with 2 ml of confluent culture as inoculate. For smoke toxicity tests, cell suspensions harvested from 5 day-old cultures were used.

### Smoke treatment

**Figure 1:**
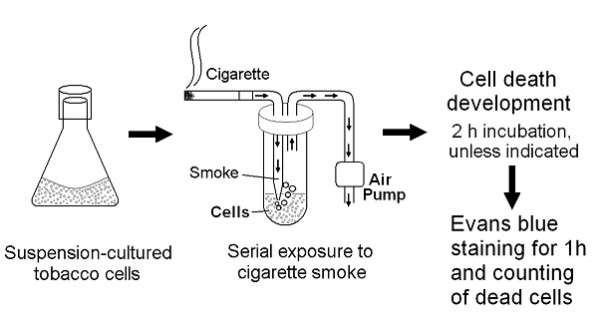
**Procedures for cigarette smoke treatment of tobacco cell suspension cultures and cell death evaluation**.

Exposure of the cell suspension to cigarette smoke was performed using a set of apparatuses illustrated in Figure 1. Cigarette smoke was obtained by lighting on the tip of cigarettes (with filters) connected to the glass pike connected to the sealed plastic test tube (50 ml) containing 10 ml of cell suspension. Cigarette smoke was loaded into the plastic tube by aspirating the air at 1.5 L/min using an air pump (HiBlow 3EBS, Kenis Kagaku Kyoeisha Ltd., Tokyo, Japan). When combustion of a cigarette was completed, additional cigarettes were used for serial exposure. Thus, the extent (dose) of smoke exposure was adjusted by the number of cigarettes used for serial exposure.

### Cell death analysis

Impact of smoke exposure was determined by monitoring the level of cell death induction according to previous reports [10]. Cigarette smoke-induced cell death in the cell suspension culture was determined by staining of the dead cells with Evans blue (0.1%, w/v) by mixing and incubating the cells and the dye for 1 h. Unless indicated, 2 h of incubation was employed prior to addition of the dye to the cells (to allow cell death development). Then stained cells were observed under microscopes (SMZ800, Nikon, Tokyo, Japan). For statistic analysis, 3-4 different digital images of cells under the microscope (each covering 50-100 cells to be counted) were acquired and stained cells were counted.

### Chemicals

Calcium chelator, 1,2-bis(2-aminophenoxy)-ethane-*N,N,N',N'*-tetraacetic acid (BAPTA) was purchased from Dojindo Laboratories, Inc. (Kumamoto, Japan). *N-*Acetylcysteine, dimethylthiourea (DMTU), 1,4-diazabicyclo[2.2.2]octane (DABCO), 6-(2-Hydroxy-1-methyl-2-nitrosohydrazino)-N-methyl-1-hexanamine (NOC-9), ethylenediaminetetraacetic acid iron (III) sodium salt (Fe(III)-EDTA), *o-*phenanthroline and Murashige-Skoog (MS) salts were obtained from Sigma (St. Louis, MO, USA). Catalase from bovine liver and other reagents of analytical grade were purchased from Wako Pure Chemical Industries (Osaka, Japan).

## Results and discussion

**Figure 2:**
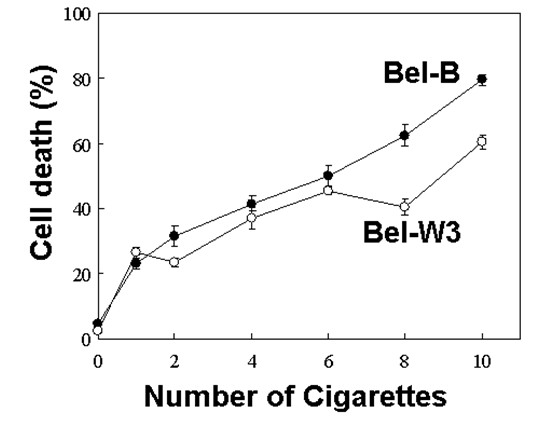
**Effect of cigarette smoke on cell death induction in two cell lines of tobacco**.

Cigarette smoke-induced cell death in two tobacco suspension-cultured cell lines was studied (Figure 2). Since Bel-B and Bel-W3 cells originally differ in the level of basal ROS production and ROS detoxification capacities, these cell lines as a pair, form a good experimental model for examining the involvement of ROS in the phenomena of interest. By exposing the cell suspensions to the cigarette smoke-containing air, we observed the gradual development of cell death in both cell lines, which depends on the dose of smoke exposure (number of cigarettes used).

Interestingly, the higher level of cell death was observed in the ROS-tolerant and detoxifying Bel-B cell line compared to Bel-W3 cell line. This implies that ROS might be involved in negative regulation of cell death development initiated by exposure to the smoke. Thus, our working hypothesis in which ROS play a positive role in cell death, was rejected, despite the reported involvements of ROS in plant cell death induced by ozone [9,10], pathogen attacks [6], and metal stress [11,12].

**Figure 3:**
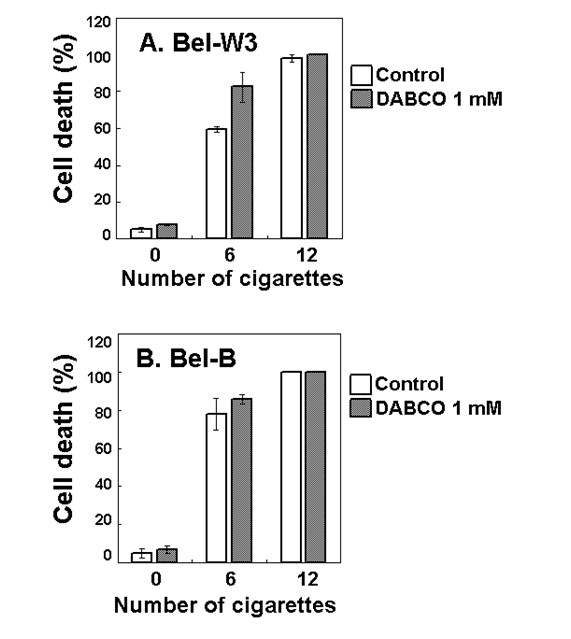
**DABCO-dependent enhancement of the cigarette smoke-induced cell death in two cell lines of tobacco**. Tobacco cells were pretreated with 1 mM DABCO, 5 min prior to smoke exposure.

**Figure 4:**
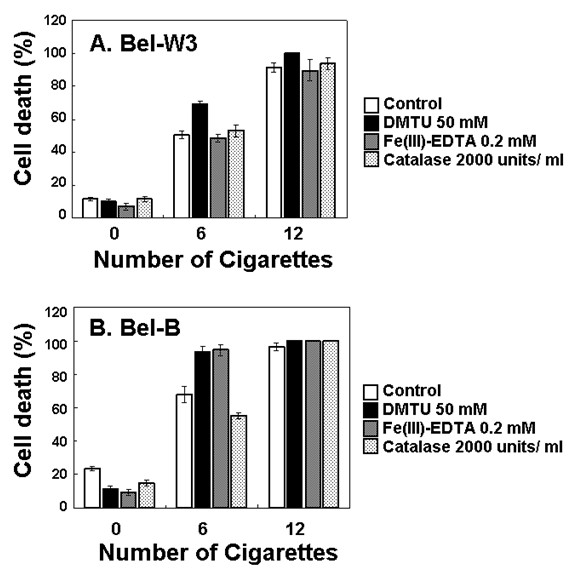
**Effect of three ROS scavengers on the cigarette smoke-induced cell death in two cell lines of tobacco**. Tobacco cells were pretreated with 50 mM DMTO, 0.2 mM Fe(III)-EDTA, or 2000 units/ml of catalase, 5 min prior to smoke exposure.

Above view was further supported by ROS-related pharmacological tests. Addition of 1,4-diazabicyclo[2.2.2]octane (DABCO, 5 mM), a scavenger of singlet oxygen, resulted in enhancement of the smoke-induced cell death in Bel-W3 cells (Figure 3). Roles of other ROS members were also examined by adding ROS scavengers namely, DMTU, a scavenger of hydroxyl radicals; Fe(III)-EDTA, a scavenger of superoxide anion radicals; and catalase which removes hydrogen peroxide (Figure 4). The smoke-induced cell death in Bel-W3 was enhanced by the presence of DMTU and that in Bel-B cells were enhanced by the presence of DMTU and Fe(III)-EDTA, suggesting that ROS (hydroxyl radicals and superoxide) are protecting the cells from smoke-induced cell death. For further confirming the role of ROS against the action of smoke, the effect of 1 mM ascorbic acid as additional ROS scavenger, enhancing the smoke-induced cell death was also observed (data not shown). Above data suggest that the actual factor(s) contributing to the cell death could be antagonistic to the action of ROS.

**Figure 5:**
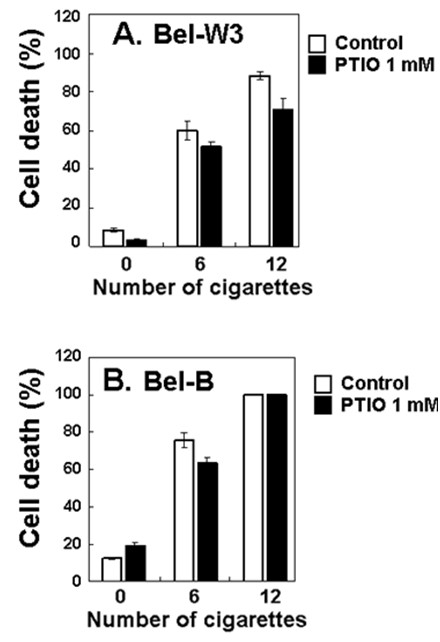
**Inhibitory effect of PTIO (NO scavenger) against the cigarette smoke-induced cell death in two cell lines of tobacco**. Tobacco cells were pretreated with 1 mM PTIO, 5 min prior to smoke exposure.

Interestingly, addition of PTIO, a scavenger of nitric oxide (NO) showed slight but significant inhibition of the cell death suggesting the role for combustion-mediated generation of NO in smoke-dependent cell death induction in plant cells (Figure 5).

**Figure 6:**
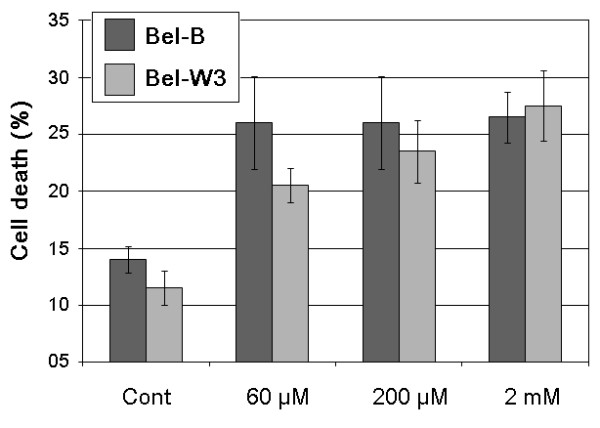
**Effect of NO donor on induction of cell death in two cell lines of tobacco**. NOC-9 (60 μM, 600 μM, and 2 mM) was added to the cell suspension and cell death was assessed after 6 h of incubation.

As PTIO showed some extent of cell protecting effect against the action of cigarette smoke, we further tested the effect of NO-generating agents on induction of cell death. By adding NOC-9 (dissolved in 0.1 N NaOH) to tobacco cells, possible role for NO in induction of cell death was examined (Figure 6). Treatment of tobacco cells with NOC-9 (60 μM - 2 mM) resulted in induction of cell death within 6 h. In case of 2 mM NOC-9, the level of cell death attained *ca*. 70% at 24 h after the treatment of both cell lines (data not shown).

**Figure 7:**
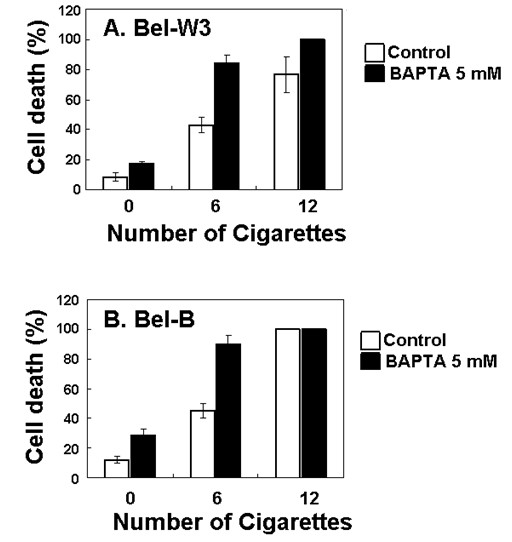
**Calcium chelator-dependent enhancement of the cigarette smoke-induced cell death in two cell lines of tobacco**. Tobacco cells were pretreated with 5 mM BAPTA, 5 min prior to smoke exposure.

Since previous studies documented that ROS actions and calcium signaling are often related each other [6,9,11-13], effect of a calcium chelator, BATPTA was also tested (Figure 7). The smoke-induced cell death was highly enhanced by addition of a calcium chelator (5 mM BAPTA). Data suggested that Ca^2+ ^is necessary required for protection of the cells from the smoke-induced cell death mechanism.

In case of smoke-induced cell death in human lung microvascular endothelial cells exposed to phosphate-buffered saline-conditioned cigarette smoke mimicking the exhaled cigarette smoke, the level of exposure was assessed by the increase in nicotine/cotinine levels, thus assessed cell injury and cell death was discussed in relation to the increase in nicotine/cotinine levels [14]. Therefore it is difficult to dissect the impact of plant-derived chemicals chiefly nicotine and the rest of chemicals formed through burning of cigarettes. In contrast, the present study proposed a novel model in which impacts of nicotine and other plant-derived chemical could be minimized or ignored. This is the key difference between plant and animal models for elucidating the mechanism behind the smoke toxicity.

There had been a trend for manufacturing cigarettes with lower nicotine yield in recent decades but at the same time there is a reported tendency for the smokers of such low-nicotine yield cigarettes to smoke more intensely and to inhale the smoke more deeply than the smoker of plain cigarettes [15]. Among the important changes in the composition of the tobacco blend of the U.S. cigarettes between 1955 and 1993, there is a significant increase in nitrate content (0.5% to 1.2-1.5%), which linearly raises the yields of nitrogen oxides (NO_x_) and N-nitrosamines in the smoke [16]. Taken together, the peripheral lungs of smokers today are likely exposed to higher amounts of NO_x _and nitrosated compounds [15,16]. Therefore, there are some increasing needs for novel cell death models focusing the impact of combustion-related gas such as NO_x_. The present model using tobacco cells (plant cell model) may fulfill such needs, thus one may expect that novel mechanism of cell death induction could be uncovered using plant cell model. However, we cannot expect any information on the chronic impact of cigarette smoke (chiefly carcinogenesis) since plant model is designed basically for acute development of cell death. In animal models, the cigarette smoke was shown to induce both chronic consequence such as cancer and acute injuries at cellular level.

Reportedly, cigarette smoke contains *ca*. 4800 identified compounds, and 69 compounds among them are carcinogens, and several are tumor promoters or cocarcinogens [17]. However, at the same time, many members of major toxic agents from cigarette smoke such as nicotine, carbon monoxide, hydrogen cyanide, NO_x_, some volatile aldehydes, some alkenes, and some aromatic hydrocarbons may behave as the causative agents for acute injuries [17].

In most works with human or animal systems at tissue levels, the mode of cigarette smoke-related injury, to be whether apoptotic or necrotic manner, could be hardly discussed. However, some cellular models (such as human spermatozoa) clearly determined that the mode of cell death induced by cigarettes smoke components to be apoptosis or programmed cell death (PCD) [18]. Also in plant cell model, we view that the cell death observed here can be considered as PCD which could be modulated through relays of cellular signaling events such as calcium signaling. Since the smoke-induced event observed here could be enhanced by blocking the calcium signaling (in the presence of BAPTA), we can conclude that the smoke-induced cell death development is regulated under active cellular signaling events, thus indicative of PCD other than necrosis. Generally in plant cell models, cytosolic Ca^2+ ^is known to be a modulator of PCD induced during various occasions (both positive and negative way) such as abiotic stress responses [9,10] and hypersensitive defense response against pathogen attacks [19,20].

Here, NO was shown to be involved as a key mediator of the smoke-induced PCD in tobacco cells as the pharmacological results with PTIO treatment (Figure 5) and NOC-9 treatment (Figure 6) clearly suggested. In plant cell model, the likely role of NO in plant cell PCD is not very conclusive. In plants, NO reportedly plays a dual role by acting both for and against PCD. In case of cadmium (Cd) toxicity in cell suspension culture of *Arabidopsis thaliana*, Cd-induced PCD was mediated with the production and action of NO [21]. In contrast, in barley aleurone layers, NO was shown to act as antioxidant thus protecting the cells from ROS-induced PCD [22]. Such antagonistic interaction between NO and ROS in induction of plant PCD was also reported during plant response to ozone [23], although other recent report did not emphasize the importance of NO during ozone-induce PCD [10]. The likely NO-ROS antagonism was also observed in our present study but the role for NO and ROS in PCD induction largely differed from the above work [22].

**Figure 8:**
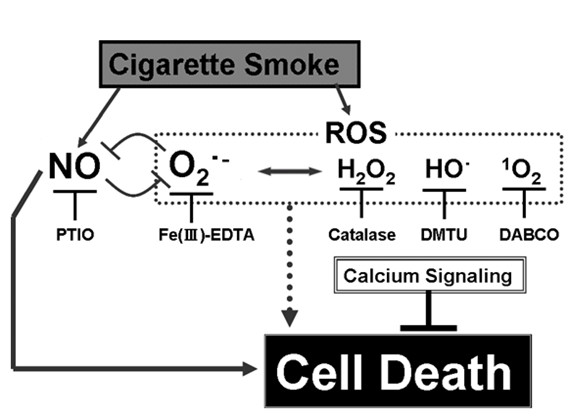
**Proposed model mechanism for cigarette smoke-induced cell death in tobacco cells**. Members of ROS may protect the cells by competing with NO. In addition to ROS, calcium is also contributing to minimizing the toxic action of NO.

Lastly, we would like to propose a novel mechanism in which the smoke-derived NO and ROS compete each other, during induction of the cigarette smoke-induced cell death in tobacco cells (Figure 8). The members of ROS, chiefly superoxide anion radical, may protect the cells from the cigarette smoke by competing with the smoke-induced NO, as superoxide is reportedly highly reactive against NO. In addition to ROS, as BAPTA enhanced the lethal action of the smoke, Ca^2+ ^can be considered as another factor protecting the living cells from the damaging impact of cigarette smoke.

## Conclusion

In conclusion, NO was identified as the key factor mediating the toxic action of cigarette smoke in tobacco cells. If this knowledge could be applicable to other biological system including human body, the effect of NO removal from the cigarette smoke must be tested for minimizing the risk of smoking.

## Competing interests

The authors declare that they have no competing interests.

## Authors' contributions

TK designed and supervised the experiments, participated in smoke treatment and cell death analysis, and drafted the manuscript. MY and TH joined the discussion, prepared the cellular materials, and participated in the cell death analysis, thus equally contributed to the present study. The authors read and approved the manuscript.
